# Validation of a method to quantify microfibres present in aquatic surface microlayers

**DOI:** 10.1038/s41598-020-74635-3

**Published:** 2020-10-21

**Authors:** Joshua Birkenhead, Freya Radford, Jessica L. Stead, Andrew B. Cundy, Malcolm D. Hudson

**Affiliations:** 1 Faculty of Environmental and Life Sciences, University of Southampton, Highfield Campus, University Road, Southampton, SO17 1BJ UK; 2 Faculty of Engineering and Physical Sciences, University of Southampton, Highfield Campus, University Road, Southampton, SO17 1BJ UK

**Keywords:** Environmental sciences, Ocean sciences

## Abstract

Many of the methods for microplastics quantification in the environment are criticised creating problems with data validity. Quantification of microplastics in the surface microlayer of aquatic environments using glass plate dipping holds promise as a simple field method, but its efficiency has yet to be validated. We tested a standard glass plate dipping method to assess recovery of four common polymer microfibres and two common natural fibres, under three different salinities (freshwater, brackish water, saltwater). Overall recovery rates were low (26.8 ± 1.54%) but higher recoveries were observed under saltwater treatments (36.5 ± 3.01%) than brackish water (24.5 ± 1.92%) or freshwater (19.3 ± 1.92%). The fibre types showed different recovery rates, with acrylic yielding significantly higher recovery rates (37.0 ± 2.71%) than other fibres across treatments. No clear relationship between the density of the fibres and the recovery efficiency was seen. We suggest that, where this method is used for monitoring microplastics, the results will typically underestimate the total amount present, but that recovery is sufficiently consistent to allow comparison of differences between sampling locations. When comparing data across river-estuarine or similar transects salinity should be monitored to account for salinity-induced differences in sampling recovery.

## Introduction

Microplastics are small plastic pieces, < 5 mm at their largest dimension^1,2^, which are considered globally ubiquitous in the marine environment^3^, with increasing evidence of their widespread presence in freshwater, terrestrial systems and the atmosphere^4^. However, there is no set method for sampling for microplastics that is in standard usage for any environmental matrix^5^. Equally, there is limited assessment of how efficient the methods in use are at extracting microplastics from a matrix, and this is mostly limited to assessment of removal from sediment, or success of identification utilising visual techniques. As a result, it is likely that microplastics are being underestimated in samples^6^.

The surface microlayer (SML) (often specifically referred to as the sea surface microlayer) is the uppermost 0–1000 µm of the ocean, and other water bodies. The SML is the link between oceans and the atmosphere, and has a number of properties that make it likely to be a zone of accumulation of microplastics. Surface tension may act to retain light, low density particles (such as microplastics) in the SML, as they already float at the surface due to their low density. The SML has also been described as ‘sticky’ due to its organic matter content^7^, which may contribute to the enriched abundance of microplastics (and other hydrophobic substances) observed in the SML as compared to underlying water^7–10^. High abundances of microplastics have been recorded in the SML, with an average value of 152,688 (± 92,384) particles m^−3^ recorded in the coastal seas of South Korea^8^. The SML forms a vital habitat for a variety of species, including commercially important species (e.g. cod larvae)^10^, which could be vulnerable to microplastic ingestion. Risks may also be increased by the interaction of microplastics with other anthropogenic pollutants which are recorded to have an enriched abundance in the SML, including persistent organic pollutants, chlorinated hydrocarbons, and heavy metals^7,11^. Therefore, an accurate assessment of microplastic abundance is necessary to fully assess the risks posed by microplastics in the SML. Previous surface water sampling methods involve the use of specialist equipment such as manta trawls or the collection of large volume bulk water which may be limited by the size range covered or the minimum sample volume required^12^. The SML is relatively simple to sample in comparison, and due to its enriched abundance of microplastics, offers potential as an effective indicator of the status of microplastic contamination in a location.

However, as with most environmental microplastic sampling, there is no standard method of sampling the SML. Methods utilised include metal sieves^8,9,13^, glass syringe^10^, sea surface microlayer collection apparatus^14^ and glass plates 7. Whilst there has been comparison of these methods in the field^7^, there has been no assessment of the recovery rate by any of these methods in terms of the percentage recovery from the SML- so such sampling, while indicating presence and general abundance, does not give an accurate indication of the actual amounts present with some form of calibration. The density ranges of different polymers, as well as buoyancy of particles and their degree of weathering and aggregation, likely affects both concentration of microplastics in the SML and their recovery. In addition, surface microlayers also exist in freshwater and brackish water environments^15^, where the less saline water has a lower (or variable) density; this may affect microplastic concentration and recovery. Previously published studies have focused on open ocean SML sampling, where salinity is likely to be less variable, however two studies have sampled the SML in estuaries, where salinity is variable, although no consideration of the effects of salinity on sampling was given^7,13^. We therefore propose that this is the first study to (a) assess recovery rates of an SML sampling method for microplastics, and (b) to consider the effects of salinity on these recovery rates.

## Results

**Figure 1:**
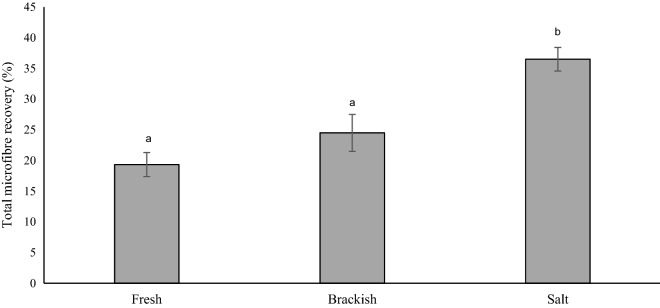
Total microfibre recovery across three different salinities: salt (33ppt), brackish (15ppt) and fresh (0.5ppt). Bars show mean (n = 15) ± SE error bars. Bars with the same letter showed no significant difference.

Recovery rates of microfibres across all salinity treatments were generally low, but reproducible (based on repeat sampling). The mean microfibre recovery across all salinities was 26.8% (± 1.54 SE, n = 15). Total microfibre recovery rate was different across the three salinities (*χ*^2^ (2) = 19.80, p = 0.000, Kruskal Wallis test) (Fig. 1). Recoveries were highest in the salt water with a mean of 36.5% (± 3.01 SE, n = 5), which was significantly higher than total microfibre recovery in brackish water (p = 0.003, Dunn’s test) with a mean of 24.5% (± 1.92 SE, n = 5), and freshwater (p = 0.000, Dunn’s test) with a mean of 19.3% (± 1.92 SE, n = 5).

**Figure 2:**
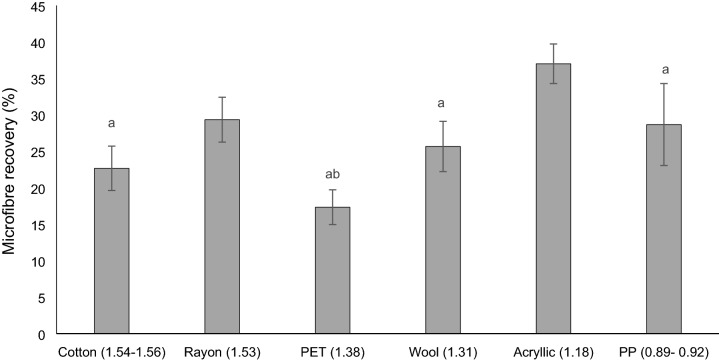
Total microfibre recovery rates with different material types (density of material is reported in brackets in g cm^−3^) ranging in natural and synthetic origin. Bars show mean (n = 15) ± SE error bars. Bars with the same letter showed no significant difference.

Microfibres were recovered at different rates in each salinity (Salt: F(5,24) = 10.44, p = 0.000; brackish: F(5,24) = 4.34, p = 006; fresh: F(5,24) = 4.20, p = 007, one-way ANOVAs). In salt water more PP, acrylic, wool and rayon microfibres were recovered than PET (p = 0.000; p = 0.000; p = 0.0.004; p = 0.004, Tukey’s tests), more PP fibres were recovered than cotton (p = 0.003, Tukey’s test) and more acrylic than cotton (p = 0.003, Tukey’s test). More acrylic fibres were recovered than PET and PP (p = 0.017; p = 0.040, Tukey’s tests) in brackish water. In fresh water more acrylic fibres were recovered than wool or cotton ones (p = 0.014; p = 0.014, Tukey’s tests). Different types of microfibres were recovered at different rates across all salinity treatments (*χ*^2^ (2) =  = 18.49, p = 0.002, Kruskal Wallis test) (Fig. 2). Acrylic had the highest recovery rates across treatments with a mean of 37.0% (± 2.71 SE). This was higher than PET (17.33% ± 2.38 SE; p = 0.000, Dunn’s test), cotton (22.67% ± 3.04; p = 0.002, Dunn’s test), PP (28.67% ± 5.61; p = 0.009, Dunn’s test) and wool (25.67% ± 3.45 SE; p = 0.013, Dunn’s test) but similar to the recovery rate of rayon (29.33% ± 3.08 SE). PET had the lowest recovery rates but was only significantly lower than acrylic and rayon (p = 0.000; p = 0.006, Dunn’s test).

**Figure 3:**
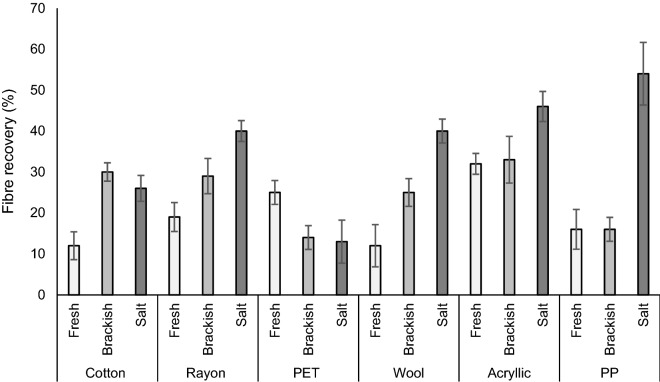
Total microfibre recovery rates with different material across three salinities (fresh, brackish and salt). Bars show mean (n = 15) ± SE error bars.

For most of the tested microfibre types, recovery was highest in salt water compared to the other salinities (Fig. 3). Significant differences were demonstrated as follows. There was higher recovery of wool in salt water compared to brackish and fresh water, and higher recoveries in brackish water compared to freshwater (F(2,12) = 22.23, p = 0.000, one-way ANOVA). PP also had higher recoveries in salt water compared to brackish and fresh water (F(2,12) = 15.96, p = 0.000, one-way ANOVA). There was higher rayon recovery in salt water compared to freshwater (F(2,12) = 7.44, p = 0.008, one-way ANOVA) and higher cotton recovery in brackish compared to fresh water (F(2,12) = 5.11, p = 0.025, one-way ANOVA).

There was no relationship between microfibre density and its recovery rate (r_s_ = − 0.16, p = 0.123, Spearman rank correlation). No fibres were found in any of the blank samples and microfibre numbers matched completely between the two counts of every sample.

## Discussion

Here we have quantified the recovery efficiency of microfibres from the SML using the glass plate method. This method has proven to be easily reproducible with added benefits of low cost and simplicity^7^. However, despite showing a high degree of sampling reproducibility (i.e. a relatively low standard error of 3% or less on n = 15 determinations) the recovery efficiency of microfibres in the test systems used was low, with an average extraction of only 26.8%. Recovery efficiency varied with salinity, and was highest in salt waters with a salinity equivalent to typical sea water. This is likely due to the variation in density of the treatment media, which accompanies changes in salinity. The denser the water, the more likely the microfibres are to become buoyant and present (or concentrated) in the SML. It is also important to consider that with increased salinity, water surface tension increases with a difference of approximately 3% between fresh and saline water^16,17^. This may contribute to the higher recovery efficiencies seen in saline water although the impact is likely to be small^17^. It is therefore recommended that future studies consider salinity when using this sampling technique to allow salinity-specific adjustments to be applied.

Microplastic sampling methodologies must be consistent, as comparing different locations sampled with different methodologies is not possible^5^. This glass plate method can be applied to a range of aquatic environments, making it easier to sample widely and to compare spatial and temporal patterns, provided that the salinity is accounted for. This will be particularly important for environments such as tidal estuaries where physical properties are constantly changing and salinity may range from 0.5 ppt to 35 ppt within one daily cycle^17,18^, and are also affected by seasonal freshwater flows, mixing and stratification, as well as tides^19^.

The variation in recovery efficiency between microfibre types must also be considered. Here we showed that recovery efficiency may be microfibre-specific. Although no overall correlation between density of material and recovery efficiency was seen, it is suspected that density plays a role in the recoveries seen here. Acrylic and PP fibres had some of the highest recoveries and are the lowest density materials we tested. Additionally, PET (which has one of the highest densities) had very low recoveries even in the salt water treatment, which is in line with previous suggestions that dense polymers tend to sink in the water column^21^, and hence were not recovered as efficiently here as the lighter polymers. However, it is probable that other physical and chemical properties also contributed to this result as the natural fibres with similar high densities did not also exhibit this pattern of recovery. Acrylic and PP are both hydrophobic with low water retention which may help to retain their low density, whereas wool has high water retention which may increase density^23^. Additionally, acrylic has moderate build-up of static which may allow for it to remain in the SML and attach to the glass plate more easily^23^. This may also explain why acrylic is one of the most dominant fibres present in environmental samples^24^. Additionally, cotton, which has the highest density, did not have the lowest recovery rates. The physical structure of cotton, which characteristically has hollow fibres with a ribbon structure, means it is prone to trapping air pockets which increase buoyancy and may overcome its high density^25^. The focus here was on fibres as they represent more than 80% of the microplastics found in the marine environment^26^. However, previous studies have found that the shape and size of microplastics may influence their sinking rate, with less spherical particles sinking at slower rates^27^. This suggests the importance of additional testing to understand the efficiency of this method for all microplastic types e.g. fragments and films. The impact of weathering and/or biofouling on microplastics may also be a consideration. Here we tested virgin microplastics, however, in the environment over a period of several months plastic debris can become negatively buoyant due to biofilm formation^28^. Weathering may also affect the surface characteristics, buoyancy and sinking behaviour of microplastics which may change the behaviour of microplastics in the SML (e.g.^27^), particularly under higher UV radiation fluxes in shallow waters and the SML.

The results presented here show the importance of considering recovery efficiencies of this sampling methodology in different salinities and for different microfibre types- and illustrates a wider issue regarding methods for isolating microplastics from environmental media where recovery rates are not known. While recovery % is sufficiently reproducible to allow comparison of bulk differences between sampling locations of similar salinities, the low recovery implies the possibility that previous studies may have underestimated the extent of microplastic contamination in the SML (depending on microplastic composition and local salinity, by a factor of 2–10). Which, considering that up to 43 fibres per litre have been found in the surface microlayer^7^ suggests the true value could be much higher. This potential under-counting has important implications when assessing the environmental risks of microplastics as exposure rates could larger than those indicated by field sampling and analysis. We suggest that future studies consider the efficiency of sampling methodologies. Here, further work may consider further the glass plate sampling method in terms of sampling quantities, differences in microplastic concentrations and particularly how it may be applied to different sizes and shapes of microplastics which may have inherently different properties^29^.

Caution should be applied however when upscaling these results as microplastics are a set of diverse environmental contaminants that are characterised by a wide variety of physical and chemical properties^30^. Additionally the SML in aquatic environments is an extremely complex system with varying physical, chemical and biological characteristics depending on seasonality^31^ and spatial distribution^32^. This is particularly important in estuarine environments where system hydrodynamics play a major role in determining the SML characteristics^33,34^, which may subsequently influence both the microplastic concentrations found there and the sampling recovery. We therefore recommend that future studies consider the application of such techniques in real environmental conditions where both the conditions of the environment and the impact they have on the physical properties of microfibres may impact sampling method efficiency. To further understand the level of microplastics contamination in the SML, the efficiency of sampling methods under these varying characteristics should be considered.

## Methods

### Microfibre materials

Microfibres were created for the purpose of this study from a range of consumer materials to represent a variety of synthetic and natural fibres likely to occur in the environment^35^. Six material types were chosen and identified from product labels: polyethylene terephthalate (PET), rayon, acrylic, wool, cotton and polypropylene (PP) (Table 1). The selection criteria for material types chosen also included the colour of the material: brightly and differently coloured materials were chosen for ease of identification. Microfibres were cut to a size of 2–5 mm to fit with current microplastic size definitions^1,2^ and to represent the natural variability likely to be found in real environmental samples^7^.

**Table 1:** Details of microfibres used in spiking experiments to include materials type, colour, original product and density^38,39^.

Material type	Density (g/cm^3^)	Colour	Original product
Polyethylene terephthalate	1.38	Black	T-Shirt
Rayon	1.53	Yellow	Cloth
Acrylic	1.18	Turqouise	Yarn
Wool	1.314	Green	Yarn
Cotton	1.54–1.56	Blue	Cotton Balls
Polypropylene	0.894–0.92	Grey	Carpet

### Glass plate dipping method

Water of three different salinities were used in the study. Tap water was used as a freshwater medium, for which the typical salinity is 0.5ppt^36^. Sodium chloride was added tap water to simulate sea water (33ppt) and brackish water (15ppt). For each of the salinities, 10 L was poured into a plastic container and spiked with 20 fibres of each of the six microfibre types (n = 120) to mimic a median of reported environmentally relevent concentrations^37^. To recover the microfibres, the glass plate method, as described in^7^, was used. Prior to the use of the glass plate, the water was throughly mixed for 30 s to ensure microfibre distribution, reduce adherence of microfibres to the edge of the container and simulate environmental disturbance.

The water was allowed to settle until there was no detectable movement, before a glass plate (148 × 210 × 5 mm) was placed in the water, perpendicular to the surface, to a depth of 180 mm. The plate was then withdrawn at a rate of 5 cm/s and the water adhered to the plate was immediately transferred to a glass beaker. This was repeated 25 times per sample, generating a volume of approximately 100 mL. The water was then filtered onto a glass microfibre filter (Whatman, GF/F, pore size: 0.7 μm) and microfibres were counted under a microscope (Nikon Optiphot, × 40). The process was repeated five times per salinity treatment. All equipment was washed thoroughly with distilled water between replicates to avoid contamination.

### Statistical analysis

All statistical analyses were performed in RStudio (1.2.1335) software. Recovery rates were determined by dividing the amount of microfibres recovered by the initial number added to give a percentage recovery. Normal distribution of data was assessed using Shapiro–Wilk tests, and homogeneity of variance with Levene’s test.

Kruskal–Wallis tests, with Dunn’s post-hoc analysis, were used to test differences in total microfibre recovery in the different salinities and recovery of each microfibre type across all salinities. One-way ANOVAs, with Tukey’s tests for post hoc analysis, were used to test differences in recovery of different microfibres in each of the salinities. A Spearman’s rank correlation was used to assess relationships between density and recovery of microfibres according to the upper limit of the density ranges provided in Table 1.

## Data Availability

Data supporting this study are openly available from the University of Southampton repository at: https://doi.org/10.5258/SOTON/D1409 .
